# Identification of the gene defect responsible for severe hypercholesterolaemia using whole-exome sequencing

**DOI:** 10.1038/srep11380

**Published:** 2015-06-16

**Authors:** Li-Yuan Sun, Yong-Biao Zhang, Long Jiang, Ning Wan, Wen-Feng Wu, Xiao-Dong Pan, Jun Yu, Feng Zhang, Lu-Ya Wang

**Affiliations:** 1 Beijing AnZhen Hospital, Affiliated to Capital Medical University, Beijing Institute of Heart, Lung and Blood Vessel Diseases. The Key Laboratory of Remodeling-related Cardiovascular Diseases, Ministry of Education, Department of Atherosclerosis, Beijing 100029, China; 2 Institute of Genomics, Chinese Academy of Sciences and Key Laboratory of Genome Science and Information, Chinese Academy of Sciences, Beijing 100101, China; 3 National Engineering Laboratory for Druggable Gene and Protein Screening, Northeast Normal University, Changchun 130024, Jilin, China; 4 Beijing AnZhen Hospital, Affiliated to Capital Medical University, Department of Dermatology, Beijing 100029, China; 5 University of Chinese Academy of Sciences, Beijing 100049, China

## Abstract

Familial hypercholesterolaemia (FH) is a serious genetic metabolic disease. We identified a specific family in which the proband had typical homozygous phenotype of FH, but couldn’t detect any mutations in usual pathogenic genes using traditional sequencing. This study is the first attempt to use whole exome sequencing (WES) to identify the pathogenic genes in Chinese FH. The routine examinations were performed on all parentage members, and WES on 5 members. We used bioinformatics methods to splice and filter out the pathogenic gene. Finally, Sanger sequencing and cDNA sequencing were used to verify the candidate genes. Half of parentage members had got hypercholesterolaemia. WES identified *LDLR* IVS8[−10] as a candidate mutation from 222,267 variations. The Sanger sequencing showed proband had a homozygous mutation inherited from his parents, and this loci were cosegregated with FH phenotype. The cDNA sequencing revealed that this mutations caused abnormal shearing. This mutation was first identified in Chinese patients, and this homozygous mutation is a new genetic type of FH. This is the first time that WES was used in Chinese FH patients. We detected a novel genetic type *of LDLR* homozygous mutation. WES is powerful tools to identify specific FH families with potentially pathogenic gene mutations.

Familial hypercholesterolaemia (FH; MIM: #143890) is an autosomal dominant hypercholesterolaemia (ADH) and is a very serious hereditary metabolic disease. The prevalence of heterozygous FH(HeFH) subjects is 1/500, and their elevated serum cholesterol levels are 2 ~ 3 times higher than those of healthy individuals. Although the prevalence of homozygous FH (HoFH) is only one per million, such patients often exhibit extreme clinical features. For example, these patients display elevated serum cholesterol levels (6 ~ 10 times higher than normal), cutaneous xanthomas, atherosclerosis, and even myocardial infarction-related death during childhood^1^. Atherosclerosis in HeFH progresses quickly, and the risk of death from coronary heart disease (CHD) is increased approximately 80-fold among FH subjects between 20 and 29 years old who have not received treatment^2^. Epidemiology studies of HeFH in France, Canada, Finland, and Lebanon showed prevalence rates of approximately 1/200, and as high as 1/137 in Denmark^3^. In 2013, the European Atherosclerosis Society (EAS) published a new consensus on the diagnosis and treatment of FH to strengthen the diagnosis and intervention for FH^4^,^5^. Recently, in Holland, scientists have proposed that the FH individual needs early screening and intervention because atherosclerosis proceeds quickly from birth^6^. Additionally, in the USA, the FH Foundation conducted a multi-centre study in 2014, including the screening and follow-up of patients with FH^7^. The early diagnosis of FH is performed worldwide.

The incidence of FH in the Chinese population is not well known because no epidemiological studies have been performed in Mainland China until now. Thus far, fewer than 100 FH cases have been reported in mainland China^8^, and even fewer in Hong Kong^9^. This phenomenon is most likely because of a lack of knowledge regarding the risks of FH and not because of a lower incidence of FH in mainland China^8^. China is the most populous country in the world; therefore, its genetic burden of FH is more serious than in other countries^9^. Our group collected samples from 50 Han patients and their families from China diagnosed as the HoFH phenotype. Through early diagnosis and intervention therapy, we are able to study the characteristics of the clinical phenotype of FH, to understand the characteristics of Chinese HoFH and HeFH patients. These data from the FH patients and their families are used to study the characteristics of Chinese HoFH and HeFH patients.

Gene mutations are the pathological basis of FH^1^. The mutant low-density lipoprotein receptor gene (*LDLR*) causes dysfunction of the LDL receptor in the cell membrane surface, which leads to the inability of the liver to remove LDL from the blood, resulting in LDL and ectopic cholesterol deposition on other organs. Many mutations were also identified in pathogenic genes, such as apolipoprotein B (*apoB*) and proprotein convertase subtilisin/kexin type 9 gene (*PCSK9*), of ADH^10^. We also identified several potential relevant pathogenic mutations in *LDLR*, *apoB* and *PCSK9* for FH^11^,^12^,^13^,^14^,^15^,^16^. These data may reveal the genetic mechanisms underlying FH and increase the knowledge of FH in the Han Chinese population.

A high-efficiency method of gene sequencing is very important for discovering pathogenic mutations. Gene mutations are undetectable in approximately 60% of the suspected autosomal dominant inheritance patients using traditional DNA sequencing method^17^. Therefore, whole genome linkage^18^ and whole genome sequencing methods have been used to screen candidate virulence genes^19^. Since 2009, the whole exon sequencing method has been used to capture exon DNA and for high-throughput sequencing, becoming the most effective strategy to identify pathogenic genes of Mendel’s disease^20^. British researchers used this strategy to identify 48 sporadic FH patients with unknown gene diagnoses, 17 of which had an *LDLR* mutation and *apoB* mutation^21^.

In our FH database, we choose a large FH family of three generations and 21 individuals from the Henan Province. The HoFH phenotype proband was 5 years old with a fasting plasma total cholesterol (TC) level of up to 21.23 mmol/L, multiple skin xanthomas and an atherosclerotic plaque in the carotid artery. However, we did not find any pathogenic gene mutations using the traditional PCR-based exon-by-exon sequencing and multiplex ligation dependent probe amplification (MLPA) method^22^. We hypothesize that this family may have a new pathogenic mutation.

## Results

### Clinical characteristics

**Figure 1:**
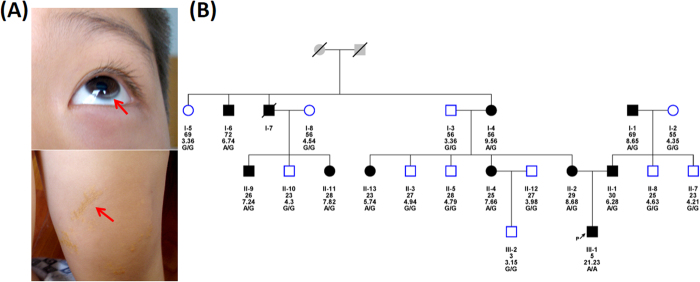
Clinical manifestations of the proband and his pedigree. (**A**) Clinical manifestations of the proband who had a typical FH phenotype: corneal arcus (up) and xanthoma planum (down). (**B**) Pedigree of the Study Family with FH. Squares indicate male family members, and circles, female family members. Slashes indicate deceased persons. The column of five values under each symbol indicates, from top to bottom, the age in years (as of 2006), the TC levels (in mmol/L) and the *LDLR* genotypes.

The proband of the FH family was born in 2001 and is from the Henan province. He had xanthomas over the Achilles tendon at 2 months of age. The xanthomas grew in size as he aged. He had a high level of lipids at 2 years of age. Physical examination revealed multiple xanthomas diffused on his hands, elbows, knees, and buttocks, particularly arcus senilis, which is often observed in the elderly (Fig. 1).

Laboratory tests revealed elevated lipid level in 8 persons of the pedigree. The TC and LDL-C of the proband were 21.23 and 19.77 mmol/L, respectively. The TC of his parents was 6.28 mmol/L and 8.68 mmol/L. The TC levels of the other 7 persons were from 5.74 ~ 8.68 mmol/L. High level TC concentrations were detected in over half the maternal branch. (Fig. 1). These biochemical tests were repeated in 2012 with no change in diagnosis.

Based on these clinical results, the proband is diagnosed as a ‘definite FH’ with a DLCN score of 16. His parents and other relatives were diagnosed as ‘probable FH’^23^.

### Whole-exome sequencing and variant calling

**Figure 2:**
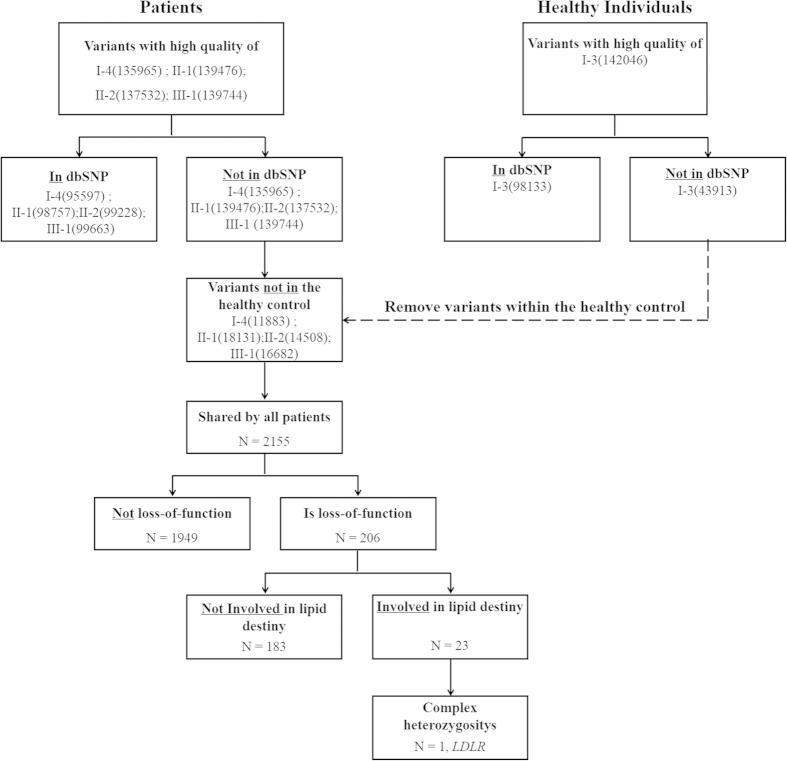
Overview of variant prioritization.

The whole exomes of I-3, I-4, II-1, II-2 and III-1 were sequenced using an NGS strategy to screen for the causal genes of the FH pedigree. The exome sequencing had a mean coverage from 32- to 58-fold among the samples and led to the detection of 190,029 SNPs, 32,107 small indels (<60-bp) and 131 structure variations. With the assumption that a causal mutation for FH is not reported or a rare variant in the known database, we removed variants with global MAF >0.01 in the database of the dbSNP138 or 1000 Genomes Project. Considering that the causal mutation is inherited in the dominant mode in the FH pedigree, we retained 2155 variants shared by patients and not detected in healthy individuals. Furthermore, the causal mutation should be lose-of-function mutation. Therefore, we focused on variants that were missense, frameshift, splicing, and conserved (GERP score >2 or phastCons score >0.8), as well as variants in transcription factor binding sites. Of these 2,155 variants, 206 were lose-of-function mutations and 23 were located in genes involved in lipid metabolism. Because the cholesterol levels of the mutant III-1 are much high than in the affected parents, we hypothesised that the mutant gene III-1 is a homozygote or complex heterozygote, with the parents as heterozygotes. Of 23 lipid-associated genes, only *LDLR* c.1187-10G>A confirmed our hypothesis (Fig. 2).

### Sequencing of LDLR within the FH pedigree, other sporadic FH patients and healthy controls

**Figure 3:**
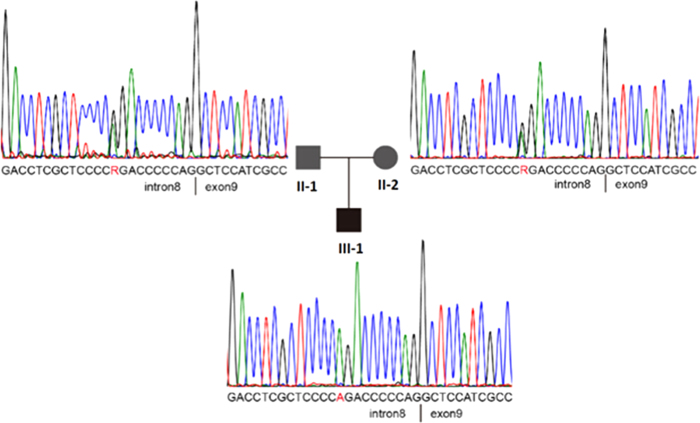
Family internal validation results showed the identification of the LDLR IVS8 [−10] (red letter).

We genotyped the coding regions and flanking regions of *LDLR* in eight high-risk patients and eight healthy individuals within this FH pedigree. This SNP was fully segregated within the FH phenotype. To ensure that *LDLR* c.1187-10G >A was specific to FH patients (Figs 1,3), we genotyped 39 sporadic FH subjects with a DLCN score greater than 8. We detected 22 mutations in these subjects but did not encounter the *LDLR* c.1187-10G >A. We also genotyped 288 healthy Chinese subjects as controls and did not encounter the mutant allele.

### *In silico* prediction of splicing alterations

We used four web-based tools to determine whether the c.1187-10G >A affected the strength of the natural splice site. ASSP, HSF, and NetGene2 identified a cryptic donor site generated by c.1187-10G >A. The quantitative or confidence scores of the cryptic donor site are higher than the natural site according to the four software tools (Table 1). The L1 distance calculated with Spliceman was 37994 with a percentile rank of 82%, which represents a high possibility that the c.1187-10G >A disrupts splicing^24^.

### Sequencing of the *LDLR* cDNA

**Figure 4:**
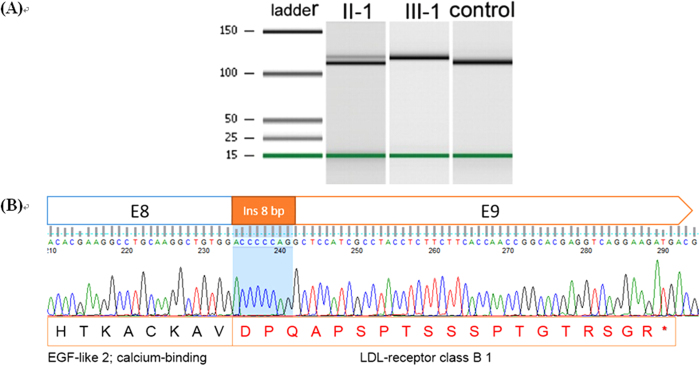
The cDNA sequence analysis of IVS 8[−10]G>A variants on mRNA splicing. (**A**) The PCR amplification products were analyzed by Agilent 2100 bioanalyzer. III-1 is the proband, II-1 is his father and control is a healthy control. (**B**) The red band shows the intron 8 area. The red capital letter represents the amino acids that would be changed by the mutation. The red band shows the eight nucleotides from intron 8. The red capital letter represents the amino acids changed by the mutation.

We genotyped the cDNA of *LDLR* in the nuclear family. The proband analyses revealed that the c.1187-10G >A activated cryptic splice sites. The mutation resulted in a transcript that included the last eight nucleotides of intron 8 in the mRNA. The normal amplified fragment was 116 bp, while the amplified fragment of II-1 showed another 124 bp fragment, and III-1 was 124 bp. The mutations modify the open reading frame, leading to an early stop codon (Fig. 4).

## Discussion

FH is not a rare disease, but many doctors and FH subjects lack awareness of this disease. Furthermore, it is underdiagnosed and undertreated in China. FH should be identified and treated as soon as possible to reduce the incidence of CHD and delay the progress of premature cardiovascular events. Therefore, we hope to define the practice of genetic testing of HoFH families to treat these patients.

In this case, we did not detect any pathogenic mutation the proband who had typical characteristics of FH and a family history of premature CHD using traditional PCR-based exon-by-exon sequencing. We attempted whole-exome sequencing and obtained over 3.5 M genome sequence variations. We excluded the majority of SNPs as nonpathogenic mutations because they also appeared in healthy individuals. Because of the autosomal dominant nature of FH disorders, the proband family alleles contained rare mutations, reducing the number of candidate genes. Finally, we located the pathogenic mutation, a *LDLR* 1187-10 G >A, a splice site mutation in the intron 8. Based on the exon sequencing, we found and confirmed one HoFH case with an inherited loci mutation - *LDLR* c.1187-10G >A in the intron 8 from his parent. We then determined that 8 subjects with high cholesterol levels also carried this mutation, but not the others who had normal cholesterol levels in the parentage. This may account for the cosegregation of the mutation with the disease phenotype.

The vast majority of ADH cases (>90%) are caused by *LDLR* mutation^25^. *LDLR* is located on Ch19p13.113.3, has a total length of 45 kb, and consists of 18 exons and 17 introns, which encode a precursor protein of 839 amino acids. The primary function of the membrane protein LDL receptor is to remove the circulating LDL by binding to and internalizing it, which are essential for the regulation of plasma cholesterol. Over 1700 *LDLR* mutations have been reported worldwide and are distributed among 18 exons, introns and promoter regions, including point mutations, such as substitutions, insertions, and deletions, and copy number variants. Each of these mutations influences the LDLR residual activities to a different degree^26^. However, many mutations that are located in introns with uncertain biological consequences have been identified as the causative mutations of FH^27^. Determining whether genetic variants are pathogenic is very important for early diagnosis, optimal treatment and further screening of family members^28^.

The mutation *LDLR* c.1187-10G >A was first identified in the Anglo-Saxon population in 2001 and has since been reported in the populations of France^29^ and the Philippines^30^. After searching the “British *LDLR* Mutation Database” (http://www.ucl.ac.uk/ ldlr/LOVDv.1.1.0/), all previously reported mutations were heterozygous, and this locus was not reported in the Chinese population^8^.

*LDLR* cDNA full-length sequencing was used to detect the proband and his family and showed that this mutation caused a 3′ splice site recognition error, shifting the eight bases of the intron to the CDS and finally leading to a frameshift mutation in the reading frame. This site is also located within the multiple polypyrimidine tract areas, which are more conservative, and it is 10 bp upstream of the natural splice site. All the predicted results from the common abnormal splice site prediction software, such as ASSP, HSF, and NetGene2, were reported as “Damage”. The cDNA sequencing revealed the insertion of eight bases, causing subsequent abnormal protein translation in the original 55 ~ 57 locations of E9, thereby generating termination codons. This process resulted in the termination of the original LDL receptor class B 1 domain at the nineteenth amino acid position.

To the best of our knowledge, this is the first use of cDNA full length sequencing of the *LDLR* gene in Chinese FH patients. Holla *et al.* reported the use of flow cytometry to analyse lymphocytes, with 1187-10G >A heterozygous patient EBV-transformed samples. The results showed that compared to the mean values of 100% in three normal cell lines, the expression of *LDLR* in 1187-10G >A lymphocytes was lower than 60%, and internalization of LDL capacity was lower than 40%. These mutations may include a degrading mutation that leads to the decay of nonsense mediated mRNA. Although the expression is not completely abolished, such truncated proteins would not be correctly located on the cell membrane because they lack the anchoring domains (exon 16 & 18). Because they cannot achieve full functionality, they appear similar to a null allele.

The highlights of this study are as follows: (1) to the best of our knowledge, this is the first study to use whole exome sequencing technology to identify gene mutations for FH in the Chinese. Our research strategy is based on routine methods, including exome trapping enrichment from the proband and three patients and one normal individual from his family, high-throughput sequencing to obtain genetic data, common software to filter out the causative genes, Sanger sequencing to validate and *in silico* tools to analyse gene splicing and function. Finally, we identified one *LDLR* c.1187-10G >A mutation. (2) This is a homozygous mutation and had been confirmed as one new mutation type after searching the UK LDLR mutation database (http:// www.ucl.ac.uk/ ldlr/LOVDv.1.1.0/). (3) This is the first study to identify the *LDLR* c.1187-10G >Amutation in Han FH patients, suggesting that it exists in population other than Caucasian.

## Methods

### Study subjects and biochemical analysis

The FH pedigree and other sporadic FH subjects were selected from the “Familial Hypercholesterolemia Sample Base of Beijing AnZhen Hospital”. The diagnosis of FH was based on the Dutch Lipid Clinic Network (DLCN) Criteria as follows: score greater than 8 for “definite FH”, ranging from 6 to 8 for “probable FH”, ranging from 3 to 5 for “possible FH”, and less than 2 for “unlikely FH”^23^. Venous blood was drawn from the antecubital vein in the sitting position after overnight fasting and was stored in tubes containing EDTA. The plasma TC, triglycerides, high-density lipoprotein cholesterol (HDL-C), and LDL-C levels were determined using a Hitachi 7600-020 automatic analyser (Hitachi, Japan). These biochemical data were retested in 2012.

This study complies with the Declaration of Helsinki and was approved by the ethics committee of the Beijing Anzhen Hospital of the Capital University of Medical Sciences. All participants provided written consent, and separate informed consent from the guardian of the proband was obtained to publish the photographs in Fig. 1.

### Whole-exome sequencing and variant calling

Within the collected FH pedigree, four affected individuals (I-4, II-1, II-2 and III-1) and one unaffected individual (I-3) were selected for whole-exome sequencing. The Agilent SureSelect Human All Exon 50 Mb kit (Agilent Technologies, Santa Clara, CA, USA) was used to capture the whole exomes. The products were resolved on an Illumina HiSeq2000 (Illumina, San Diego, CA, USA). Paired-end sequencing with a 100-bp read length was conducted on each sample. All paired reads were mapped to the human reference genome (hg19) using BWA (version 0.7.5a-r405). PCR duplication of the reads was removed using Picard (version 1.92). Samtools (version 0.1.19) and GATK (version 2.7) were used to call the variants the within genome-wide region instead of within the 50-Mb targeted region^31^,^32^. Pindel was used to detect the structural variations^33^. Finally, 190,029 SNPs, 32,107 small indels (<60-bp), and 131 structure variations were obtained.

The variants were annotated with SeattleSeq Annotation 138 and were sequentially filtered with the following criteria: (1) remove the variants with a global minor allele frequency greater than 0.01 in the database for dbSNP 138 or 1000 Genomes Project; (2) retain the variants consistent with model of dominant disease transmission; (3) keep the loss-of-function variants, such as missense, frameshift, splicing, and conserved (GERP score >2 or phastCons score >0.8), as well as variants in transcription factor binding sites; and (4) keep the variants that passed manual confirmation using the IGV package.

### Sequencing of LDLR within the FH pedigree, sporadic FH patients and healthy controls

We sequenced the coding regions and their flanking regions of LDLR gene for all members of the FH pedigrees and 288 unrelated healthy controls using the Sanger sequencing method. The sequencing reactions were performed using Applied Biosystems Big Dye Terminator chemistry, and the products were resolved on ABI Prism 3730XLDNA Analyzers (Applied Biosystems, Carlsbad, CA, USA). The sequence trace files were analysed using the Phred/Phrap/Polyphred/Consed (University of Washington, Seattle, WA, USA) software. The base-quality value threshold was set to 20in Phred, with a 99% probability that the base is accurate, and all polymorphic sites were manually inspected by at least two individuals.

### RNA isolation, PCR amplification, cDNA synthesis and sequencing

The total RNA was isolated within 2 ~ 6 h from the samples collected in EDTA tubes. The RNA was isolated using the QIAamp RNA Mini Blood Kit (Qiagen, USA) following standard protocols. The cDNA was synthesized using the First Strand cDNA Synthesis Kit (Protoscript TM, NEB, USA), according to the manufacturer’s instructions. The RNA was eluted in RNase-free water and quantified spectrophotometrically on a NanoDrop ND-1000 Spectrophotometer (Mapu Scientific LLC, China). RT-PCR was performed using 0.5 μg total RNA, 0.6 μM primers and the Qiagen One-Step RT-PCR Kit (Qiagen, German). The primers and the conditions for the thermal cycling were set to amplify the five overlapping regions. The PCR paired primers were 5′ CTGGAGGGTGGCTACAAGTGC 3′ and 5′ GTGCCGGTTGGTGAAGAAGAG 3′. The products of PCR were analyzed by Agilent 2100 bioanalyzer (Agilent Technologies, USA).

### Computational prediction of splicing alterations

We used four *in silico* tools, the Alternative Splice Site Predictor (ASSP)^34^, Human Splice Finder (HSF)^35^, NetGene2^36^, and Spliceman^37^ to predict the impact of C.1187-10G >A on the pre-mRNA splicing of *LDLR*. These software tools provide quantitative or confidence scores for the wild-type and mutant splice site sequences that reflect the strength of the splice site. ASSP, HSF, and NetGene2 identify the most likely splice site in a given sequence, and then assign a score for the splice site strength. We considered a mutation deleterious to normal splicing if the score of the cryptic splice site was greater than the natural splice site. Spliceman calculates a L1 distance and a percentile rank to measure the effect of a point mutation on splicing. A greater L1 distance and percentile rank indicate that a point mutation disrupts splicing. All parameters and score thresholds were set to the default recommendation for each software.

## Additional Information

**How to cite this article**: Sun, L.-Y. *et al.* Identification of the gene defect responsible for severe hypercholesterolaemia using whole-exome sequencing. *Sci. Rep.* **5**, 11380; doi: 10.1038/srep11380 (2015).

## Figures and Tables

**Table 1:** The predictive strength of the acceptor site of the cryptic (cgctccccagACCCCCAGGC) and natural (agacccccagGCTCCATCGC) sequences.

**tools**	**quantitative/confidence score**	
	**natural acceptor**	**cryptic acceptor**
NetGene2	0.45	0.96
ASSP	5.702	6.093
HSF	86.26	89.65
